# Amyloids in synthetic applications

**DOI:** 10.1038/s41598-022-25499-2

**Published:** 2022-12-08

**Authors:** Marianna Török

**Affiliations:** Department of Chemistry, University of Massachusetts Boston, 100 Morrissey Blvd., Boston, MA 02125 USA

**Keywords:** Protein aggregation, Biophysical chemistry, Synthetic chemistry methodology, Biomaterials - proteins, Nanoparticles

## Abstract

More than a century after the first description of amyloids by Alois Alzheimer, and despite the enormous research efforts since then, the field is still full of surprises. While searching for answers to questions for example on the driving force, mechanism, and regulation of amyloidogenesis, or on the structure, physiological and pathological roles of different amyloid aggregates, unexpected properties are regularly revealed, broadening their application possibilities. This Collection aims to focus on the beneficial sides of amyloid formation, primarily exploring the potential use of amyloids in material science, bioengineering, and synthetic chemistry.

Amyloids are versatile multifaceted self-assembled nanomaterials. Alois Alzheimer described them first in his 1907 paper as insoluble plaques found in the postmortem brain of a dementia patient. He named these ‘amyloids’ referring to carbohydrates, but later the deposits were proven to primarily consist of proteins^1,2^. Since then misfolded amyloid-like protein deposits have been identified as hallmarks of several human diseases. Of these, the aging related Alzheimer’s disease (AD) remained the most extensively studied as it affects the largest population of patients and represents dramatically increasing personal and societal problems. The controversial amyloid hypothesis has been both widely used and a frequently debated model in AD research^3,4^. Meanwhile, new amyloids appear everywhere; more recently amyloidogenic sequences of the viral SARS-CoV-2 spike protein were identified and hypothesized as a potential contributing factor to long COVID-19 symptoms in the ongoing pandemic^5^. In addition, amyloidogenesis, the formation of these highly ordered protein aggregates with common physicochemical properties, seems to be a general feature of all polypeptides including even common globular proteins^6^. Recently, amyloid-like assemblies of simple metabolites have also been reported^7^.

While pathological amyloids are being associated with a growing number of diseases; non-pathological forms also appear in many organisms performing a wide range of normal physiological functions ranging from hormone storage to structural support and defense mechanisms^6,8^. In addition, self-assembly of peptides gains a growing interest in synthetic chemistry, bioengineering and material science as these aggregates can serve as a simple, low cost, green, and tuneable catalysts of chemical reactions or scaffolds and building blocks for nano/biomaterials. The highly stable, ordered, and customizable amyloid aggregates can be applied as templates to produce various nanomaterials or used as biomimetic catalysts^9–11^. Despite the extensive efforts and vast amount of results reported on amyloids, especially on those formed from peptides/proteins of biomedical importance (e.g., amyloid-β, tau, α-synuclein, prions, insulin), the structural characterization of the intermediates/fibrils and understanding the driving force, mechanism, or modulation opportunities of the self-assembly in a particular amyloidogenic system remained challenging. The transient nature of the intermediates, branched, nonlinear aggregation pathways, and polymorphism of fibrils are just a few examples of the many arising complications^12–16^. These difficulties somewhat limit widespread synthetic applications in practice at present.

The following papers in this Collection give a glance at recent research efforts trying to solve the mysteries of amyloid formation and explore new, exciting features that could broaden the scope of future applications. It includes papers that focus directly on potential applications of amyloids as well as those with rather biology/disease-oriented emphasis that shed light on properties that could be exploited by follow-up studies. For example, Vergunst and Langelaan^17^ identified structural features influencing the self-assembly of hydrophobins, small natural proteins secreted by fungi, that could be functionalized into commercial green emulsifiers or surface modifying agents for health-, food- or other industries. Another interesting application of engineered amyloid curli fibers from *E. coli* was presented by Saldanha et al.^18^. Using amyloids as scaffolds fused with a pH-responsive fluorescent protein, and trapping in a textile matrix, the resulting biosynthesized amyloid-based textile-composites can be used as potential wearable pH sensor skin patches for healthcare and fitness industries. The amyloidogenic 21-29 fragment of β_2_-microglobulin (β_2_m_21–29_) was proposed by Tseng et al.^19^ as a potential framework for functional biopolymers that could alternate between parallel and anti-parallel β-sheet structures in response to pH-changes. Biverstål and co-workers^20^ developed a generic approach using Bri2 BRICHOS-based fusion proteins to decorate amyloid fibrils with functional proteins for applications in cell culture, tissue engineering, drug delivery, etc. Finally, Schiattarella et al.^21^ aimed to decipher the origin of the unexpected intrinsic photoluminescence exhibited by amyloid-like systems.

As illustrated by all these articles among others, amyloids are ubiquitous and highly customizable. While biomedical, structural, and mechanistic investigations will remain important in the field, future developments will likely be directed toward more catalytic and material science applications.
